# Orbital Complications After Cochlear Implant Surgery in a Patient With Silent Sinus Syndrome

**DOI:** 10.7759/cureus.64724

**Published:** 2024-07-17

**Authors:** Tawfiq Khurayzi, Walaa H Algadhi, Hassan Ghafiry, Khalid T Ardi, Isra Aljazeeri

**Affiliations:** 1 Otolaryngology - Head and Neck Surgery, Cochlear Implant Center, King Fahad Central Hospital, Jazan, SAU; 2 Otorhinolaryngology - Head and Neck Surgery, King Abdullah Ear Specialist Centre (KAESC) King Abdulaziz University Hospital, King Saud University, Riyadh, SAU; 3 Surgery, College of Medicine, Jazan University, Jazan, SAU; 4 Otolaryngology - Head and Neck Surgery, King Fahd Central Hospital, Jazan, SAU; 5 Otolaryngology - Head and Neck Surgery, King Faisal Medical City, Abha, SAU; 6 Otolaryngology, Cochlear Implant Center, Aljaber Hospital, Ahsa, SAU

**Keywords:** maxillary sinus, hearing loss, orbital cellulitis, cochlear implant, silent sinus syndrome

## Abstract

Cochlear implantation is an effective procedure for treating patients with severe to profound sensorineural hearing loss. Silent sinus syndrome (SSS) is an uncommon disease that affects the maxillary sinus. It is diagnosed clinically and confirmed radiologically. This study describes the case of a four-year-old child who presented with bilateral profound congenital hearing loss with a family history of congenital hearing loss. The patient had no significant complaints regarding the paranasal sinuses or orbits. Radiological evaluation, including temporal bone computed tomography (CT) and magnetic resonance imaging (MRI) of the ear and internal auditory meatus, showed normal anatomy of the inner ear and petrous bone bilaterally. However, findings of SSS were incidentally detected in the left maxillary sinus. The patient underwent bilateral simultaneous cochlear implantation. On the second postoperative day, he developed left-sided ophthalmoplegia, pain on eye movement, mild proptosis, and upper and lower eyelid swelling with erythema and tenderness. The patient improved rapidly following antibiotic treatment and was almost normal by the fifth postoperative day with no notable findings; hence, he was discharged. Surgeons should carefully evaluate preoperative radiological images of the paranasal sinuses for any malformation or pathology, so that appropriate medical or surgical treatment can be given.

## Introduction

A cochlear implant (CI) is an auditory sensory prosthesis that is used to improve hearing in patients with severe to profound sensorineural hearing loss, and is designed to replace the function of the inner ear.

In 1990, the United States Food and Drug Administration (FDA) approved cochlear implantation in children from the age of two years, whereas recently the FDA approved the procedure in children aged one year onwards [1].

The cochlear implantation procedure comprises a cortical mastoidectomy with drilling of the facial recess, followed by a round window approach or cochleostomy approach to insert the electrode array. Additionally, a bony bed seat for the implant is drilled by some surgeons in the skull bone under the temporalis muscle. During any part of the surgery, injury to the adjacent anatomical structures can lead to unwanted complications.

Silent sinus syndrome (SSS) is a rare syndrome which is usually noticed as a spontaneous, painless, unilateral disorder that affects the maxillary sinus [2]. Occasionally progressive enophthalmos and hypoglobus occur due to the gradual collapse of the orbital floor with maxillary sinus opacification in the presence of subclinical maxillary sinusitis [3]. The exact etiology of SSS is not known but it may result from ostium occlusion, interruption of normal sinus development through the first or second decade of life, and formation of a destructive cyst [4]. It is diagnosed clinically and confirmed radiologically [5].

In sinus diseases and surgeries, orbital complications, such as periorbital ecchymosis, orbital cellulitis, cavernous sinus thrombosis, and subperiosteal and intraorbital abscess are more common in children compared to adults [6]. It is very rare for patients to present with orbital involvement post-CI surgery. There are few reports in the literature regarding these complications of cochlear implantation. Preoperative radiologic evidence of rhinosinusitis, positioning of the patient during surgery, and length of surgery may be at risk of orbital sequelae after cochlear implantation. A study conducted among children who had received bilateral CI demonstrated that the age of the children with orbital complications ranged from 1.1 to 7.6 years, with a mean age of 3.9 years [6].

In this study, we report the orbital complications in a patient diagnosed with SSS after undergoing CI surgery. We also reviewed the literature for the reported cases of orbital complications and sinus diseases post-cochlear implantation.

## Case presentation

A four-year-old child presented with bilateral hearing loss since birth, with no benefit from a hearing aid, along with a positive family history of congenital hearing loss. The parents reported no symptoms suggestive of paranasal sinus or orbital diseases. On examination, non-syndromic craniofacial morphology was observed and the ears were unremarkable bilaterally. Auditory brain stem response (ABR) test showed bilateral non-identifiable ABR waves at 85 dbnHL, reflecting severe to profound sensorineural hearing loss (SNHL) in the frequency range of 2-4 KHz of the audiogram.

Radiology

Temporal bone computed tomography (CT) and magnetic resonance imaging (MRI) were performed as per routine pre-cochlear implantation protocols, and the temporoparietal bone, including the inner ear and petrous bone bilaterally showed no abnormalities.

**Figure 1:**
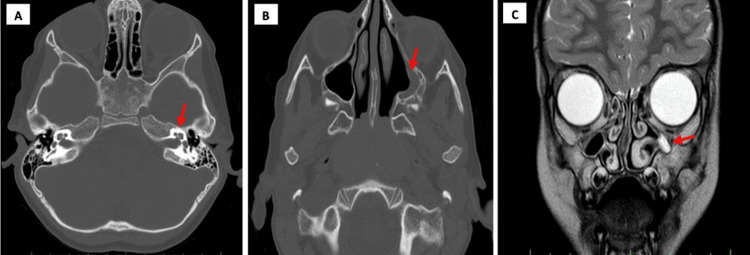
(A) Axial high-resolution computed tomography (HRCT) of the temporal bone. (B) Axial CT of paranasal sinuses. (C) Coronal magnetic resonance imaging (MRI) (A) Axial high-resolution computed tomography (HRCT) of the temporal bone showing normal bony inner ears and petrous bone on both sides. (B) Axial CT of paranasal sinuses showing normal right maxillary sinus with retraction and shrinking of the left maxillary sinus with rudimentary mucosa in the lateral wall. (C) Coronal magnetic resonance imaging (MRI) showing fully opacified left maxillary sinus with thickening and enhancement of the mucosa.

An incidental finding indicative of SSS in the left maxillary sinus was observed in both the CT and MRI. As shown in Figure 1, the MRI finding was compatible with the CT finding.

The patient underwent bilateral simultaneous CI surgery. The surgical procedure started with a post-auricular incision of 3 cm in length just posterior to the postauricular sulcus. Following fascial dissection superior to the temporoparietal fascia, a superiorly based mucoperiosteal flap was elevated. Then, the subperiosteal flap was elevated to create a tight pocket for the internal receiver stimulator. As is routine in our institute, cortical mastoidectomy was performed, followed by a posterior tympanotomy. Electrode array insertion was done through the round window. The same process was performed in both ears, and the surgery was smooth with no difficulties or significant bleeding. Intraoperative audiological measurements and post-operative X-ray showed appropriate intracochlear placement of the electrode array. The incision was closed in three layers and a loose mastoid dressing was applied and retained till the day after the operation.

On the first postoperative day, the patient was well without any complaints. Physical examination was unremarkable. On the second postoperative day, the patient developed left-sided ophthalmoplegia, pain on eye movement, mild proptosis, and upper and lower eyelid swelling with minor erythema and tenderness. These features were compatible with orbital cellulitis. The patient stayed in the hospital for monitoring and medical treatment. Intranasal saline irrigation with decongestant nasal spray and intravenous antibiotics consisting of a combination of clindamycin and ceftriaxone were administered. The patient improved rapidly and was almost normal by the fifth postoperative day with no notable findings on physical examination of the face and orbit. Hence, the patient was discharged. Two weeks later, the patient was evaluated again in the Outpatient Department and was found to be doing fine with a completely normal facial and orbital examination.

## Discussion

In this report, we have described the rare complication of orbital involvement after cochlear implantation in a patient with SSS. Cochlear implantation is generally considered a safe procedure in children, and the incidence of complications is low [6]. Orbital complications are rare after cochlear implantation.

Few studies have reported the etiology of orbital complications following ear surgery. The first explanation for periorbital edema relies on the passage of the periorbital vascular supply through the surgical field. The superficial temporal and internal maxillary vessels, which supply the periorbital area, travel on the deep surface of the temporal muscle and temporoparietal fascia. Any intraoperative injury to these vessels may lead to disruption of the blood flow, resulting in extravasation of the fluid in the thin layer of connective tissue between the skin of the eyelid and the underlying muscular layer [7,8].

The other proposed explanation is that the superficial muscular aponeurotic system (SMAS) in the otological surgical field and periorbital area is devoid of fat and has limited retaining ligaments compared to the midface area. This anatomical difference might predispose to easier fluid retention and migration of any lymphatic extravasation from the temporal to the periorbital area [8,9].

An injury to these vessels and the lymphatic drainage can occur either due to direct intraoperative injury or during postoperative pressure dressing. An intraoperative injury is most likely to occur during fascial dissection of the areolar tissue deep to the auricular muscles and superficial to the temporalis fascia, especially if a difficulty is encountered in identifying the right plane or in going further anteriorly.

Another proposed etiology for orbital complications is an infective process or rhinosinusitis. Transmission of infection from the nose to the eye could be the result of a long operation or placement of the facial nerve monitor probe in the periorbital area. Additionally, it is possible that the transmission of the vibration force during mastoid drilling could cause minor trauma to the dehiscence of the lamina papyracea and increase bacterial load in the periorbital area [6].

Some arguments against this theory exist, including that in most of the reported cases, periorbital edema occurred on the first day after surgery, similar to the present case. Therefore, it is unlikely for any infective process to occur during this short duration. Furthermore, the orbital presentation is mostly self-limiting [6].

SSS is a rare disease in which there is an asymptomatic collapse of the maxillary sinus resulting in the inward retraction of the sinus wall. The patient may present with a progressive facial deformity in the form of enophthalmos, hypoglobus, medial displacement of the globe, or proptosis [10,11].

Periorbital edema and orbital cellulitis have not been associated with SSS.

SSS is rarely diagnosed in children, and it is usually missed due to difficulty in recognition and late presentation after the development of facial deformity [5]. Most reported cases were patients in their 3rd to 5th decade of life [4,12]. It is characterized by the radiological absence of the maxillary sinus [5]. Nasal endoscopy and clinical evaluation are essential to diagnose suspected cases of SSS, while CT is the gold standard because it highlights the bony structures [5,13]. CT helps to assess and evaluate the sinuses to exclude or confirm SSS, and shows maxillary opacification, sinus wall retraction, and thinned or absent orbital floor [5]. Furthermore, CT of the temporal bone is an important part of the pre-cochlear implant surgery assessment. It can help in detecting a pathology or anomaly of the paranasal sinuses during routine preoperative evaluation of patients undergoing CI surgery.

Although there is no clear association between SSS and periorbital edema or cellulitis, it can be assumed that the patient in this study had an anomalous vascular or lymphatic drainage due to SSS that increased the risk of post-CI fluid retention in the periorbital area.

Even though the existing literature suggests that post-CI periorbital edema is a self-limiting condition with a non-infective etiology, aggressive antibiotic treatment was given to this patient. This was because transmission of the infection to the site of the surgery can lead to implant biofilm formation, which is an extremely difficult condition to treat, often necessitating an explantation and re-implantation [14].

## Conclusions

Postoperative orbital complications might occur in patients undergoing cochlear implantation surgery with radiological evidence of paranasal sinus anomalies. Although an infective etiology for orbital complications in the earliest days of the postoperative period is unlikely, prompt medical treatment might be advisable to avoid exacerbation of the infection and involvement of the implanted device. Surgeons performing cochlear implantation should take adequate steps to control intraoperative bleeding and minimize the drilling and elevation of soft tissues and fascia, particularly in the temporoparietal area.
